# Density Functional Theory (DFT) Study of Edaravone Derivatives as Antioxidants

**DOI:** 10.3390/ijms13067594

**Published:** 2012-06-20

**Authors:** Rosivaldo S. Borges, Auriekson N. Queiroz, Anna P. S. Mendes, Sanderson C. Araújo, Luiz C. S. França, Edna C. S. Franco, Walace G. Leal, Albérico B. F. da Silva

**Affiliations:** 1 Amazon Nucleus of Studies and Selection of Biomolecules, Institute of Health Sciences, Federal University of Pará, 66075-110, Belém, PA, Brasil; E-Mails: auriekson@gmail.com (A.N.Q.); annamendes@ufpa.br (A.P.S.M.); francalcs@ldschurch.org (L.C.S.F.); 2 Postgraduate Program in Neuroscience and Cell Biology, Institute of Biological Sciences, Federal University of Pará, 66075-110, Belém, PA, Brasil; E-Mails: sanderson@gmail.com (S.C.A.); ednacristinafranco@gmail.com (E.C.S.F.); wgomesleal@gmail.com (W.G.L.); 3 Institute of Chemistry of São Carlos, University of São Paulo, CP 780, 13560-970, São Carlos, SP, Brasil; E-Mail: alberico@iqsc.usp.br

**Keywords:** edaravone derivatives, ionization potential, bond dissociation energies, antioxidant, DFT

## Abstract

Quantum chemical calculations at the B3LYP/6–31G* level of theory were employed for the structure-activity relationship and prediction of the antioxidant activity of edaravone and structurally related derivatives using energy (*E*), ionization potential (IP), bond dissociation energy (BDE), and stabilization energies (Δ*E*_iso_). Spin density calculations were also performed for the proposed antioxidant activity mechanism. The electron abstraction is related to electron-donating groups (EDG) at position 3, decreasing the IP when compared to substitution at position 4. The hydrogen abstraction is related to electron-withdrawing groups (EDG) at position 4, decreasing the BDE_CH_ when compared to other substitutions, resulting in a better antioxidant activity. The unpaired electron formed by the hydrogen abstraction from the C–H group of the pyrazole ring is localized at 2, 4, and 6 positions. The highest scavenging activity prediction is related to the lowest contribution at the carbon atom. The likely mechanism is related to hydrogen transfer. It was found that antioxidant activity depends on the presence of EDG at the C_2_ and C_4_ positions and there is a correlation between IP and BDE. Our results identified three different classes of new derivatives more potent than edaravone.

## 1. Introduction

**Figure 1:**
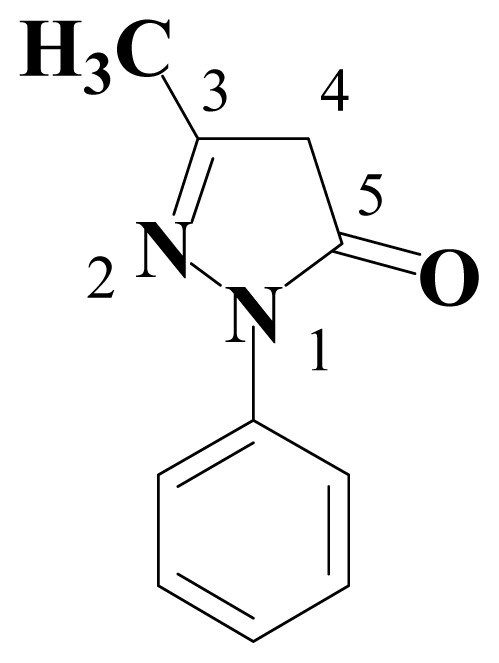
Chemical structure and numbering of edaravone.

Edaravone or 3-methyl-1-phenyl-2-pyrazolin-5-one, EDV (Figure 1), is a novel neuroprotective agent that was approved for the acute therapy of embolic stroke, and has great potential to protect against toxicity induced by various radicals [1]. The neuroprotective effect of edaravone has been evaluated in several models of cerebral ischemia and two studies have reported that treatment with this compound reduces the increase of hydroxyl radical and superoxide anion levels induced after ischemia [2,3]. The pharmacological effect of EDV arises from its radical-scavenging activity. In fact, it is efficient to scavenge hydroxyl radical (HO^•^) and DPPH radical (DPPH^•^) [4]. Therefore, EDV scavenges DPPH radical through donating the H-atom at position 4. Their strong radical-scavenging activity results mainly from 2-pyrazolin-5-one and substituents have little influence on the activity, which provides new clues to modify EDV to give better antioxidants [5].

We were encouraged to study the structure-activity relationship (SAR) as a means of characterizing the structural features of EDV and optimizing the structure with regard to its radical scavenging activity. For this purpose, we have studied the tautomerism influence on the antioxidant activity of EDV [6,7].

**Figure 2:**
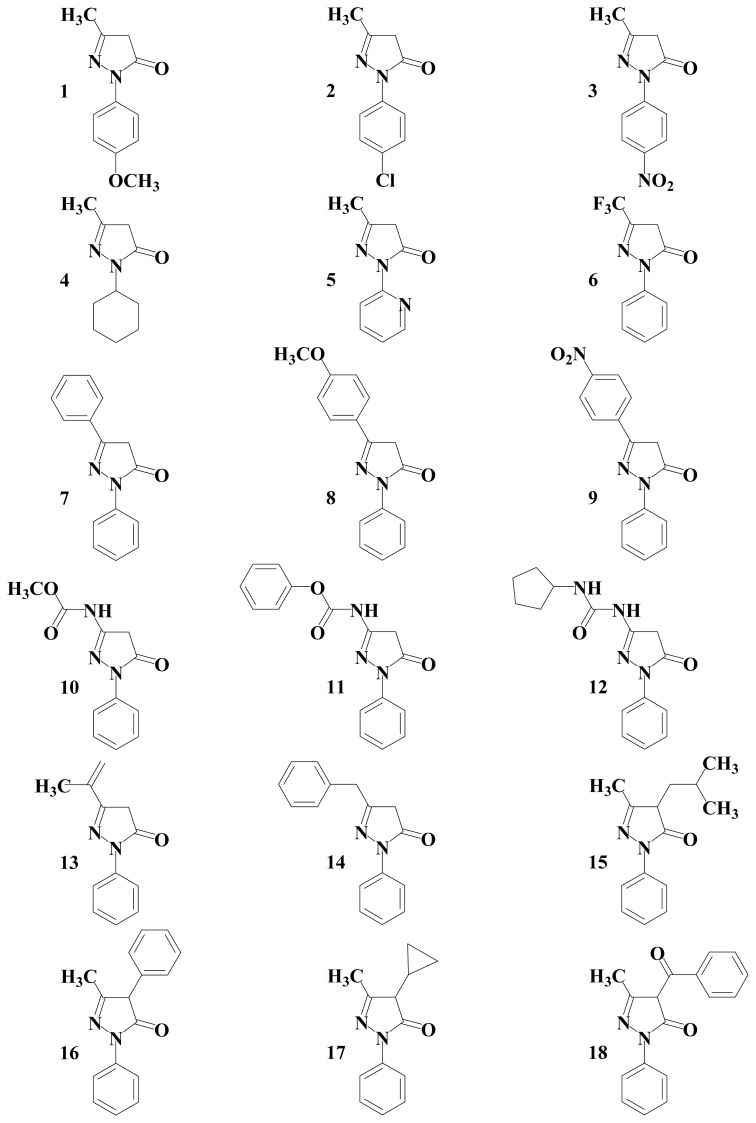
Chemical structure and numbering of edaravone.

Now, we have shown the structure-activity relationship of EDV derivatives with various substituents such as electron-withdrawing groups (EWG), electron-donating groups (EDG), and π-conjugated groups at 1-, 3-, or 4-positions of the pyrazolone ring (Figure 2), using ionization potential and bond dissociation energy of various edaravone-related derivatives, and analyzed their characteristics as free-radical scavengers under several mechanisms. Nonetheless, previous works have indicated that lipophilic substituents were essential to show its lipid peroxidation-inhibitory activity [5]. Therefore, our purpose here is to contribute to a better understanding of the mechanistic features of these processes and in the development of drugs that can be more active than edaravone.

## 2. Results and Discussion

The stabilization energy (Δ*E*_iso_) is used as simple method to predict the ability of antioxidants to trap free-radicals of edaravone derivatives. In Table 1, Δ*E*_iso_ values are shown and according to these values, it is possible to establish the following relative stability for the radicals at specified positions: the introduction of a methoxy group, an electron donating group, at the *para* position in the phenyl ring increases the Δ*E*_iso_ due to the fact that more ether moieties can donate electrons to stabilize the semiquinone form. By the way, an addition of chlorine or nitro groups as electron withdrawing group (EWG) at the *para* position in the phenyl ring decreases Δ*E*_iso_ and lowers the scavenging capacity.

The replacement of phenyl to cycle-hexanyl at the 1-position of the pyrazolone ring increases Δ*E*_iso_. Nevertheless, the change of phenyl to heterocyclic pyridinyl at the 1-position of pyrazolone ring decreases Δ*E*_iso_.

In general, substitution in the 3-position of the pyrazolone ring using an amide group increases Δ*E*_iso_, except for molecule **12**. The same result was observed for a trifluoromethyl group. On the other hand, other alkyl substitutions in this position decrease Δ*E*_iso_.

The phenyl ring or alkyl groups connecting at the 4 position in the pyrazolone ring may stabilize the radical formed during oxidation, extending the conjugation via resonance effects and contributing to the increase of Δ*E*_iso_. In fact, these compounds have the highest Δ*E*_iso_ values. In contrast, benzoylation at the 4-position in the pyrazolone ring does not seem to be important for Δ*E*_iso_.

As a consequence, molecules that showed several resonance structures were more stable and have higher Δ*E*_iso_ values. The substitution in the 1 and 3 positions does not permit good electronic conjugation between the pyrazolone ring and other groups. Nonetheless, molecules substituted in the 4-position with electron donating-groups (EDG) are characterized by a great electronic conjugation and the Δ*E*_iso_ values depend mainly on the pyrazolone ring and EDG in the 4-position.

These results agree with physical tests since oxidation potentials of the edaravone and pyrazolone derivatives were measured by cyclic voltammetry in an aqueous solution [5]. In fact, during redox process, the derivatives with strong EWGs had relatively higher oxidation potentials. On the contrary, the derivatives with strong EDGs had relatively lower oxidation potentials. Other derivatives showed a wide variety of oxidation potentials regardless of the electronic properties of the substituents (Table 1). It is possible that the oxidation potentials were not only affected by the electron density on the pyrazolone ring but also by the stability of the resulting radical species with conjugated π-orbitals on the substituent or for the reason why these molecules have a poor solubility in water. In comparison, among the positions of the substituents, the substitution at positions 1 and 3 did not positively affect the reduction of the oxidation potential, whereas substitution at position 4 seemed to be more effective.

Consequently, the compounds with higher Δ*E*_iso_ values will have lower reduction potential values. In fact, during the oxidative process, the conjugation and electronic delocalization depends on the position and EDGs in resonance stabilization, especially in the case of alkyl or phenyl ring.

**Figure 3:**
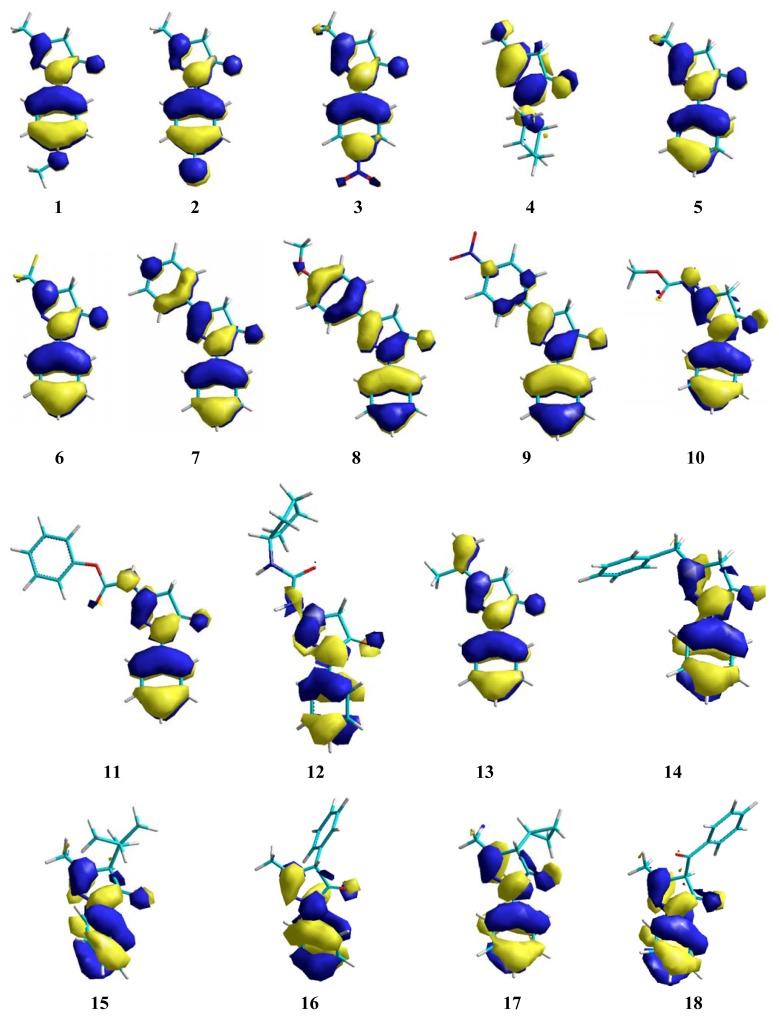
HOMO conformation of edaravone and its derivatives.

**Figure 4:**
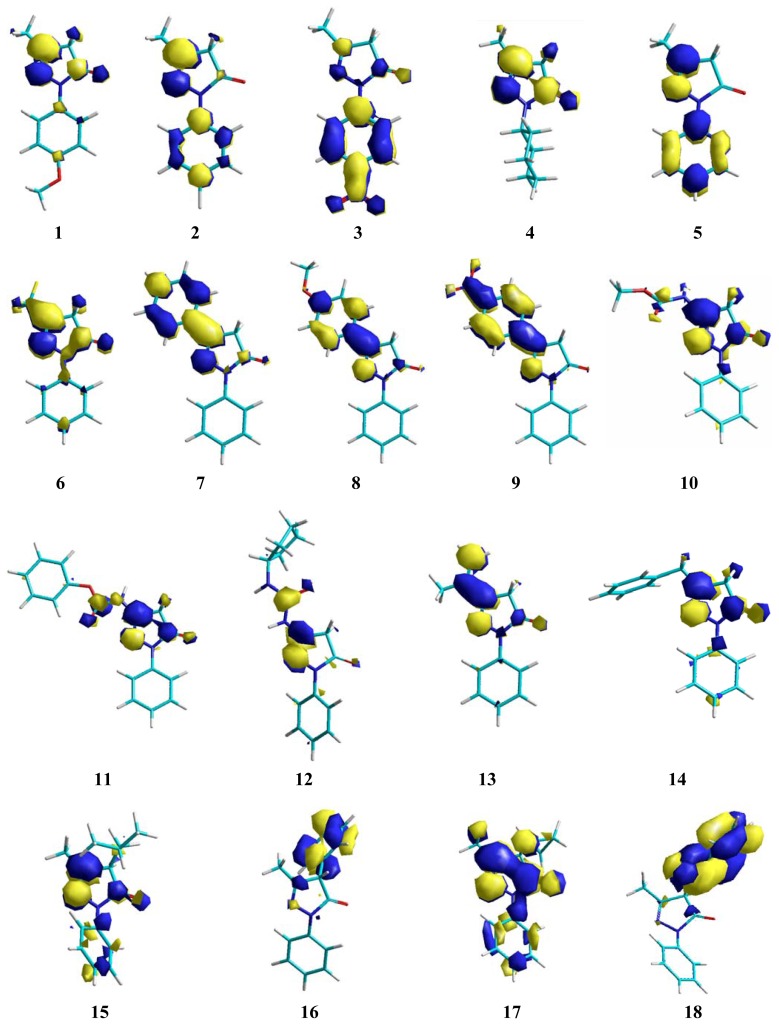
LUMO conformation of edaravone and its derivatives.

The influence of EDGs on nucleophilicity of these compounds is greater in the 4-position of the pyrazolone-ring. Since inductive and resonance effects have good participation over π conjugation between all substituents, this behavior can be increased by participation of other substituents at the 4-position. These resonance effects can be observed in Figures 3,4.

**Figure 5:**
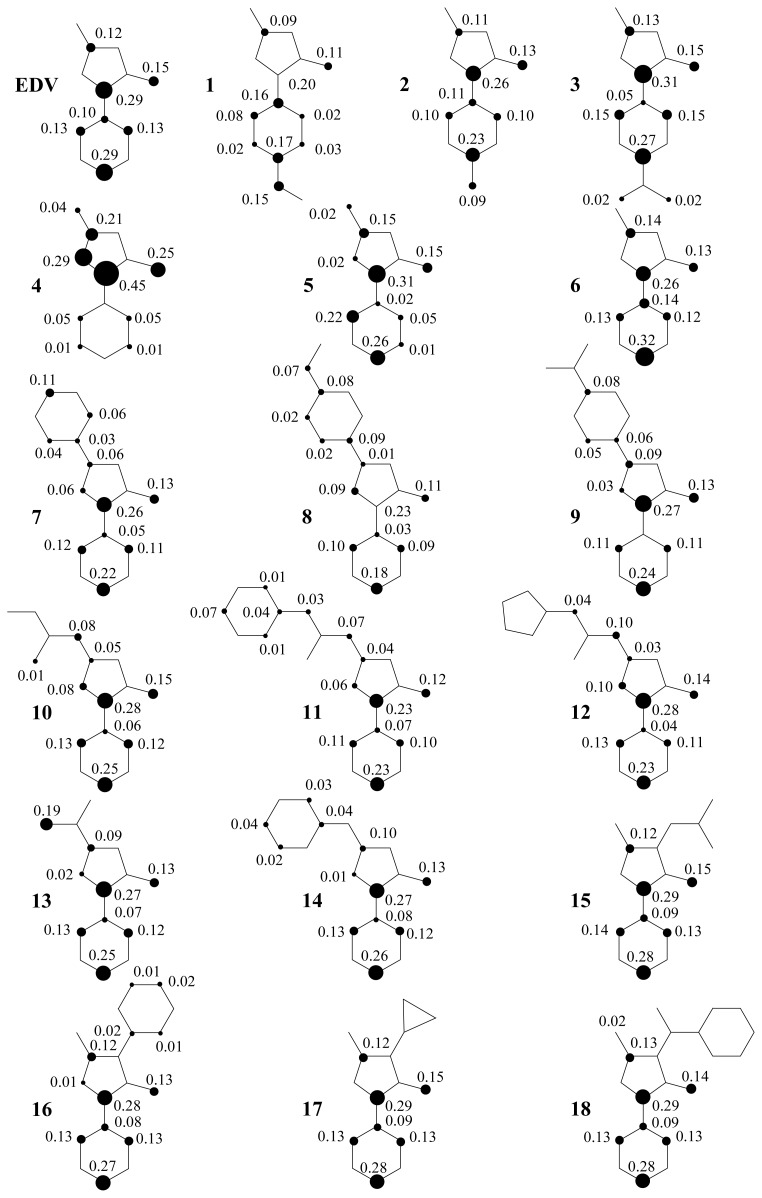
Spin density of cation free-radical of edaravone and its derivatives.

**Figure 6:**
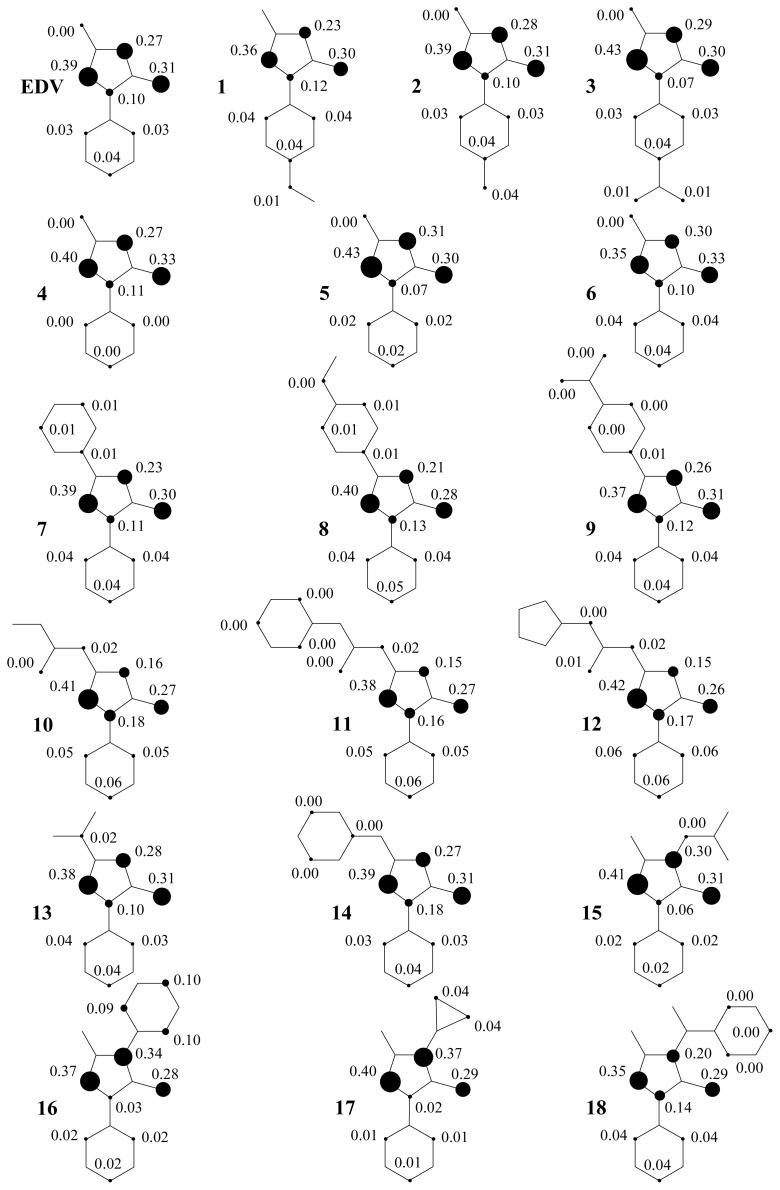
Spin density of semiquinone free-radical of edaravone and its derivatives.

Nevertheless, an alkyl or aryl moiety at the 3-position is important for conjugation over the pyrazolone ring. In fact, this is observed in compounds **4**, **5**, **7**, **8**, and **13**. The additional contribution of the phenyl ring is important for better scavenging activity, as shown in compound **8**. The planar orientations are affected by the extension of the π system changing the energy in accordance with alkyl or aryl groups at the 3-position. Thus, an alkyl or aryl substituted at the 3-position of pyrazolone ring and EDG has more influence over ionization potential (IP) values, as showed in Table 1. These results are correlated with calculated spin densities due to electron abstraction, where these substituents showed the highest contributions (Figures 5,6).

The gas-phase BDE_CH_ for edaravone has been determined in previous theoretical studies [4] using the DFT/B3LYP method, with the value being 77.93 kcal mol^−1^. The IP value of 7.50 eV is also in agreement with the experimental result, 8.00 eV [8]. Values for bond dissociation energy of methylene group (BDE_CH_) are shown in Table 1.

Nonetheless, we have observed slight agreement between ionization potential and experimental methods for compounds with high antioxidant activity. However, the introduction of EDG in 1, 3 or 4 positions decreases the IP values when compared with edaravone, resulting in a better antioxidant activity. The introduction of EWG increases the IP values. These results suggest that for these compounds the hydrogen transfer mechanism is preferred for the scavenging process mediated by physical methods, such as cyclic voltammetry.

In fact, the introduction of an EDG at the 4-position (molecules **15**, **16** and **17**) decreases BDE_CH_, resulting in an increase of the antioxidant activity when compared to edaravone.

In addition, we have observed a slight agreement between HOMO and experimental methods for compounds with high antioxidant activity. However, the better antioxidant activity does not depend on the nucleophilicity or electrophylicity.

Pérez-González and Galano have shown the importance of ionization of C–H bond in the redox capacity of edaravone [9,10]. Furthermore, our experimental studies using DPPH and ABTS methods [11] have demonstrated that the scavenging activity of edaravone and its related analogues follows a different pattern by electron or hydrogen abstraction. It is clearly seen that the scavenging activity of edaravone and phenylbutazone is significantly higher than for antipyrine and dypirone.

These results showed that molecules that have *sp*^3^ carbon atoms are significantly more active than those that have *sp*^2^ carbon atoms at 4-position of the pyrazolone ring. Therefore, our results have confirmed that the edaravone make the oxidation reaction by hydrogen abstraction in accordance with other works [4,5,9,10].

In fact, our theoretical results show lower BDE_CH_ values when phenyl ring or alkyl groups are connected to the 4 position in the pyrazolone ring in accordance with cyclic voltammetry. For these compounds, the molecules with BDE_CH_ lower than 82 kcal·mol^−1^ are more active than edaravone, while molecules with BDE_CH_ higher than 82 kcal·mol^−1^ are less active than edaravone. However, Nakagawa *et al*. [5] have found that edaravone derivatives with values of 227–275 mV (molecules **15**–**17**) are more actives. In agreement with experimental results, our theoretical BDE_CH_ values for these molecules are lower than 76 kcal·mol^−1^.

Therefore, maybe the effectiveness of edaravone derivatives depends not only on the stability of the pyrazolyl radical formed in the reaction, but also on the substituents at different positions with respect to the pyrazolone ring. For example, alkyl or aryl groups at the 4 position related to the pyrazolone ring can stabilize a carbon radical by inductive and resonance effects due to the electron-deficient radical site.

In Figure 5, the calculated spin densities of the hydrogen abstraction from the methylene group showed a contribution at carbon C_4_ of 35–43%, 26–33% for oxygen of carbonyl moiety, and 2–18% for O_6_ and the N_1_ of hydrazide moiety. The lowest contributions in these positions are related to the highest scavenging activity prediction. In addition, alkyl and aryl moieties have the highest spin density contributions only when we have the substitution at position 4.

The spin density is an important parameter to characterize the stability of free-radicals, since the energy of a free-radical can be efficiently decreased if the unpaired electrons are highly delocalized through the conjugated system after hydrogen abstractions [12–18]. This agrees with Wang [4] who states that a possible explanation for the potential antioxidant activity of edaravone might be found in the possible stabilization of the radical that is formed after hydrogen abstraction.

The prevalent contributions of BDE_CH_, Δ*E*_iso_, and spin densities are determinants for the biggest stable free-radical and more resonance structures, and show that the π-type electron system of pyrazolone derivatives substituted with EDGs at 4-position is the major modification related to the increase of the antioxidant activity of edaravone. Therefore, we clarify the possible link between hydrogen transfer antioxidant activity of edaravone due to the lowest contributions of HOMO and IP, and the highest contributions of BDE_CH_ and Δ*E*_iso_.

## 3. Computational Methods

In this paper, all structures were submitted initially to a conformational search using the PM3 method [19]. In addition, geometry optimizations of edaravone and related derivatives have been carried out using density functional theory (DFT) [20], for the reason of its excellent compromise between computational time and description of electronic correlation. The calculations were performed with the Gaussian 03 molecular package [21]. One hybrid functional of the DFT method, which consists of the Becke’s three parameters exact exchange functional (B3) combined with the non local gradient corrected correlation functional of Lee-Yang-Parr (LYP), denoted B3LYP [22,23] is used with 6–31G* basis sets [24,25] for the DFT geometry optimizations. The optimized structures were confirmed to be real minima by frequency calculation (no imaginary frequency). For the species having more conformers, all conformers were investigated. The conformer with the lowest electronic energy was used in this work. The ionization potential (IP) was calculated as the energy difference between the cation free-radical and the respective neutral molecule (Equation (1)).

(1) $$\text{IP}=[{\text{EPyr}}^{•+}]-\left[\text{EPyr}\right]$$

The bond dissociation energies (BDE) of the C–H group and its formation were calculated as the energy difference between the neutral molecule and the respective semiquinone plus hydrogen radical (Equation (2)).

(2) $$\text{BDECH}=[{\text{EPyr}}^{•}+{\text{EH}}^{•}]-[\text{EPyrH}]$$

The radical stability was determined by the calculation of stabilization energies (Δ*E*_iso_), as shown in Equation (3) for the hydrogen transfer, where the edaravone derivatives are represented by Pyr and the edaravone molecule is represented by EDV.

(3) $$\Delta \text{Eiso}=[{\text{Pyr}}^{•}+\text{EDVH}]-\left[\text{PyrH}+{\text{EDV}}^{•}\right]$$

The theoretical study of eighteen different edaravone derivatives was realized (Figure 2). We have therefore undertaken a systematic study of the influence of the alkyl or aryl substituted by EWG or EDG on the antioxidant activity of edaravone derivatives. To this aim, we have calculated: (i) the highest occupied molecular orbital (HOMO); (ii) ionization potential (IP); (iii) bond dissociation energy (BDE); (iv) Stabilization energy; and (v) spin density. These values are correlated to the experimental values of scavenging activity of the edaravone derivatives obtained with cyclic voltammetry by Nakagawa *et al*. [5]. The theoretical values are showed in Table 1.

## 4. Conclusions

In this work, the antioxidant prediction of edaravone derivatives was investigated theoretically at the DFT/B3LYP level of theory. The electron-donating groups (EDGs) at 1, 3, or 4 positions have great importance in the resonance stabilization. The relative stability of the radical forms depends on specific positions, as alkyl or aryl groups at 4-position, and contributes to the resonance effect. The electron abstraction is related to EDGs at position 3, decreasing the ionization potential (IP) when compared to substitutions at position 4. The hydrogen abstraction is related to EDGs at position 4, decreasing the BDE_CH_ when compared to other substitutions, resulting in a better antioxidant activity. Our results showed that the hydrogen abstraction is more related to the scavenging activity of edaravone and its derivatives.

## Figures and Tables

**Table 1:** Theoretical properties of edaravone and its derivatives.

Compound	HOMO (eV)	IP (kcal mol^−1^)	BDE_CH_ (kcal mol^−1^)	Δ*E*_iso_ (kcal mol^−1^)
**EDV**	−5.73	173.0	82.1	0
**1**	−5.27	161.0	81.3	−0.8
**2**	−5.86	174.2	82.4	0.3
**3**	−6.44	187.5	83.3	1.2
**4**	−6.00	183.0	80.6	−1.4
**5**	−6.01	179.8	82.8	0.7
**6**	−6.22	184.7	81.0	−1.0
**7**	−5.65	165.7	82.0	−0.1
**8**	−5.44	158.9	82.1	0
**9**	−6.06	174.8	81.8	−0.3
**10**	−5.54	166.6	81.9	−0.2
**11**	−5.59	165.8	81.8	−0.3
**12**	−5.42	162.4	82.5	0.4
**13**	−5.71	169.6	83.9	1.8
**14**	−5.73	170.1	82.6	0.5
**15**	−5.69	170.4	75.9	−6.1
**16**	−5.71	169.8	73.3	−8.8
**17**	−5.73	171.5	74.5	−7.6
**18**	−5.73	173.0	83.8	1.7
